# Robotic versus Open Radical Cystectomy: An Updated Systematic Review and Meta-Analysis

**DOI:** 10.1371/journal.pone.0121032

**Published:** 2015-03-31

**Authors:** Leilei Xia, Xianjin Wang, Tianyuan Xu, Xiaohua Zhang, Zhaowei Zhu, Liang Qin, Xiang Zhang, Chen Fang, Minguang Zhang, Shan Zhong, Zhoujun Shen

**Affiliations:** Department of Urology, Ruijin Hospital, Shanghai Jiaotong University School of Medicine, Shanghai, P. R. China; Carolina Urologic Research Center, UNITED STATES

## Abstract

**Objective:**

To critically review the currently available evidence of studies comparing robot-assisted radical cystectomy (RARC) with open radical cystectomy (ORC).

**Methods:**

A comprehensive review of the literature from Pubmed, Web of Science and Scopus was performed in April 2014. All relevant studies comparing RARC with ORC were included for further screening. A pooled meta-analysis of all comparative studies was performed and publication bias was assessed by a funnel plot.

**Results:**

Nineteen studies were included for the analysis, including a total of 1779 patients (787 patients in the RARC group and 992 patients in the ORC group). Although RARC was associated with longer operative time (p <0.0001), patients in this group might benefit from significantly lower overall perioperative complication rates within 30 days and 90 days (p = 0.005 and 0.0002, respectively), more lymph node yields (p = 0.009), less estimated blood loss (p <0.00001), lower need for perioperative and intraoperative transfusions (p <0.0001 and <0.0001, respectively), and shorter postoperative length of stay (p = 0.0002). There was no difference between two groups regarding positive surgical margin rates (p = 0.19).

**Conclusions:**

RARC appears to be an efficient alternative to ORC with advantages of less perioperative complications, more lymph node yields, less estimated blood loss, lower need for transfusions, and shorter postoperative length of stay. Further studies should be performed to compare the long-term oncologic outcomes between RARC and ORC.

## Introduction

Radical cystectomy (RC) and pelvic lymph node dissection (PLND) are the standard treatments for muscle invasive and high risk non-muscle invasive bladder cancer [1]. Open radical cystectomy (ORC) is a procedure that has been troubled with high rates of perioperative complication and mortality [2]. Minimally invasive surgeries, such as laparoscopic radical cystectomy (LRC) and robot-assisted radical cystectomy (RARC) have been applied increasingly with the goal of decreasing perioperative morbidity and mortality. Menon et al. [3] reported the first series of RARC in 2003 and demonstrated its safety and feasibility. Since then, especially in the latest 5 years, RARC has gained its popularity and achieved decent long-term oncologic outcomes [4,5]. As to comparative analysis of complication rates and perioperative outcomes between RARC and ORC, different studies showed controversial results [6–20]. In 2012, Li et al. [21] conducted a systematic review of literatures, with a meta-analysis of the results to compare RARC with ORC. The analysis of complication rates in their meta-analysis, however, were relatively sketchy because the authors did not take fully account of complication grades and postoperative period. Besides, some high-quality studies, including a randomized controlled trial (RCT), have been reported since 2012 [22–25]. Therefore, we performed an updated meta-analysis of literatures comparing complication rates and perioperative outcomes of RARC with those of ORC.

## Methods

### Search strategy and study selection

A literature search was performed in the electronic databases of PubMed, Web of Science, and Scopus. Language was restricted to English. The following terms and their combinations were searched in [Title/Abstract]: cystectomy, bladder resection, robotic, robot, robot-assisted, and da Vinci. The last updated search was performed on April 10, 2014. Article selection proceeded according to the search strategy based on Preferred Reporting Items for Systematic Reviews and Meta-analysis criteria [26]. Cited references from the selected articles were manually searched and assessed. The following inclusion criteria were used: (i) studies comparing RARC with ORC; (ii) at least one of the quantitative outcomes were included; and (iii) RCT, prospective or retrospective comparative study design. Review articles, case reports, editorials, comments, letters to the editor, and conference abstracts were excluded.

### Data extraction and outcomes of interest

Two reviewers (X. W. and T. X.) independently extracted and summarized the following data from the included studies: authors, publication year, country, study design, matching factors (age, gender, body mass index, American Society of Anesthesiologists score, diversion type, clinical stage, Charlson index, neoadjuvant chemotherapy, previous abdominal/pelvic radiotherapy, previous pelvic/abdominal surgery, and numbers of surgeon), and outcomes of interest. The outcomes of interest were perioperative complication rates within 30 days or 90 days of the date of surgery and other perioperative outcomes. Complications were classified into grade 1, 2, 3, 4, and 5 according to the Clavien-Dindo grading system [27]. Other perioperative outcomes were positive surgical margin (PSM; including urethral/ureteric and soft tissue PSM) rates, lymph node yields (LNY), operative time (OT), estimated blood loss (EBL), transfusion (including perioperative and intraoperative transfusion) rates, and postoperative length of stay (LOS). Any disagreements were resolved by discussion until a consensus was reached.

### Study quality assessment

The level of evidence (LOE) of included studies was rated according to the criteria provided by the Oxford Center for Evidence-Based Medicine [28]. The methodological quality of the studies was assessed using Newcastle-Ottawa Scale (NOS) for observational comparative studies [29] and Jadad scale for randomized controlled trials (RCTs) [30,31]. The NOS evaluates the quality of studies by examining three aspects of the study design: patient selection, comparability of the study groups, and assessment of outcomes. A score of 0 to 9 may be given to individual studies. Studies achieving a score of 7 or more indicate a high quality. Jadad scale is a 5-point scale and a score of 2 or less indicates low quality while 3 or more high quality. Two reviewers (X. W. and T. X.) independently assessed the quality of the studies and disagreement was resolved by consensus.

### Statistical analysis

The meta-analyses were performed using Review Manager Version 5.2 (The Cochrane Collaboration, Software Update, Oxford). The weighted mean difference (WMD) and odds ratios (ORs) were used to compare continuous and dichotomous variables, respectively. All results were reported with 95% confidence intervals (CIs). For studies presenting continuous data as median and range or interquartile range, the means and standard deviations (SDs) were calculated using the methodology described by Hozo et al. [32] in keeping with Cochrane handbook [33]. Statistical heterogeneity between studies was assessed using the chi-squared (χ^2^) test with a P value of <0.1 considered to indicate statistical significance, and heterogeneity was quantified using the inconsistency (I^2^) statistic. A random-effect model was used for outcomes that displayed significant heterogeneity with I^2^ values >50%; otherwise, the fixed-effect model was used. To evaluate the difference in results from studies with surgeon have greater experience in RARC, subgroup analysis of studies with more than 50 RARC cases was also performed. The outcomes of subgroup analysis include overall complication rates within 30 days and 90 days, PSM rates, LNY, OT, EBL, and LOS. Publication bias was assessed by a funnel plot.

## Results

### Characteristics and methodological quality of included studies

**Fig 1:**
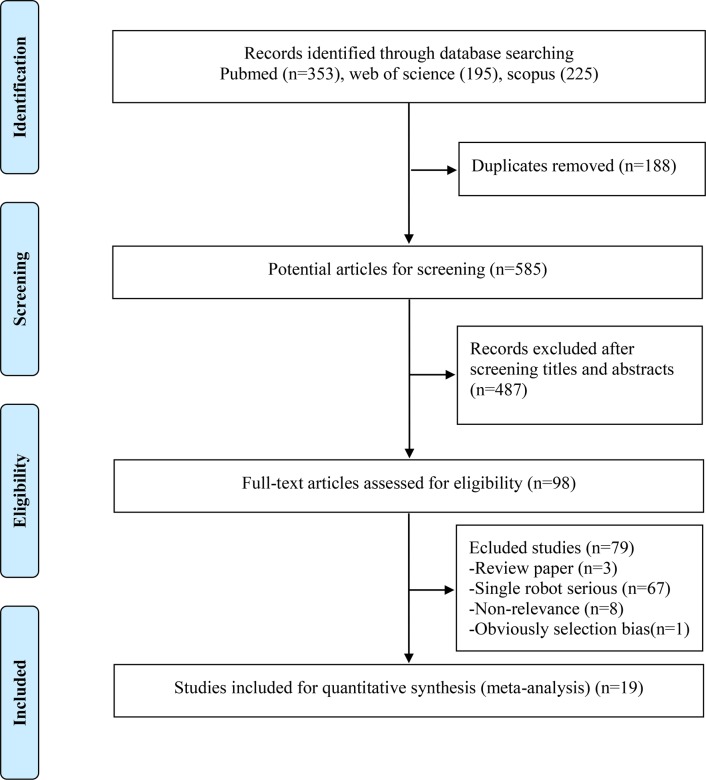
Flowchart for records selection process of the meta-analysis.

The literature search yielded 585 studies, of which 19 were selected in the final analysis including 1779 cases (787 cases for RARC and 992 cases for ORC) (Fig 1) [6–19,22–25]. Three [6,9,12] and another three [8,10,25] publications shared overlapping populations but had some different outcomes. The characteristics of included studies are summarized in Table 1. As for urinary diversion, sixteen adopted the extracorporeal method [6,7,9–17,20,22–25]. Similar neobladders were reconstructed between RARC and ORC groups (131 of 593 vs 125 of 654 in thirteen studies [6,7,9–14,16,17,23–25] (p = 0.08).

**Table 1:** Characteristics of the included studies and quality assessment.

First author, year of publication	Country	Study design	LOE	Quality Scores*	No. of patients		Urinary diversion method	No. of neobladder		Matching^#^
					RARC	ORC		RARC	ORC	
Galich 2006 [6]	USA	Prospective	3	6 of 9	13	24	Extracorporeal	5	7	1, 2, 3, 5, 6, 11
Pruthi 2007 [7]	USA	Retrospective	3	7 of 9	20	24	Extracorporeal	10	5	2, 5, 6, 11
Sterrett 2007 [8]	USA	Prospective	3	7 of 9	19	33	Unclear	NA	NA	1, 2, 3
Wang 2008 [9]	USA	Prospective	3	7 of 9	33	21	Extracorporeal	12	5	1, 3, 4, 5, 6, 9, 10, 11
Ng 2010 [10]	USA	Prospective	3	6 of 9	83	104	Extracorporeal	26	29	2, 3, 4, 5, 6, 7, 11
Nix 2010 [11]	USA	RCT	2b	2 of 5 points	21	20	Extracorporeal	7	6	1, 2, 3, 4, 5, 6
Richards 2010 [12]	USA	Retrospective	3	7 of 9	35	35	Extracorporeal	3	4	1, 2, 3, 4, 5, 6, 8, 9, 10
Martin 2011 [18]	USA	Prospective	3	6 of 9	19	14	Unclear	NA	NA	1, 2, 3, 4
Abaza 2012 [19]	USA	Prospective	3	7 of 9	35	120	Unclear	NA	NA	1, 5, 6, 8
Gondo 2012 [13]	Japan	Prospective	3	6 of 9	11	15	Extracorporeal	4	6	1, 2, 3, 5, 7, 8, 9,10, 11
Khan 2012 [14]	UK	Prospective	3	7 of 9	48	52	Extracorporeal	6	5	1, 2, 4, 5
Styn 2012 [15]	USA	Prospective	3	7 of 9	50	100	Extracorporeal	NA	NA	1, 2, 3, 4, 5, 6, 7, 8, 9, 10
Sung 2012 [16]	Korea	Retrospective	3	7 of 9	35	104	Extracorporeal	22	19	1, 2, 3, 4, 6, 7, 8, 9, 10
Kader 2013 [25]	USA	Retrospective	3	7 of 9	100	100	Extracorporeal	3	12	1, 2, 3, 4, 5, 8, 10
Knox 2013 [24]	USA	Retrospective	3	7 of 9	58	84	Extracorporeal	5	9	1, 2, 3, 4, 5, 6, 8, 9,10
Musch 2013 [23]	German	Prospective	3	7 of 9	100	42	Extracorporeal	22	7	1, 2, 3, 4, 6, 7,
Nepple 2013 [17]	USA	Retrospective	3	7 of 9	36	29	Extracorporeal	6	11	3, 5, 6, 8, 11
Parekh 2013 [22]	USA	RCT	2b	2 of 5 points	20	20	Extracorporeal	NA	NA	1, 2, 3, 4, 6, 8, 10, 11
Ahdoot 2014 [20]	USA	Retrospective	3	7 of 9	51	51	Extracorporeal	NA	NA	Propensity adjusted

RARC = robot-assisted radical cystectomy; ORC = open radical cystectomy; LOE: level of evidence; RCT = randomized controlled trial; NA = data not available.

*Using Newcastle-Ottawa Scale (NOS) for observational comparative studies and Jadad scale for RCTs.

^#^Matching: 1 = age; 2 = gender; 3 = body mass index; 4 = American Society of Anesthesiology score; 5 = diversion type; 6 = clinical stage; 7 = Charlson index; 8 = neoadjuvant chemotherapy; 9 = previous abdominal/pelvic radiotherapy; 10 = previous abdominal/pelvic surgery; 11 = numbers of surgeon

There were two RCTs comparing the two procedures [11,22] (evidence level: 2b). Ten observational studies declared prospective data collection [6,8–10,13–15,18,19,23] and seven were retrospective studies [7,12,16,17,20,24,25]. All observational comparative studies had evidence level 3. Thirteen of them had a score of ≥ 7 and were considered high quality.

### Meta-Analysis Results

#### 1. Complications

**Fig 2:**
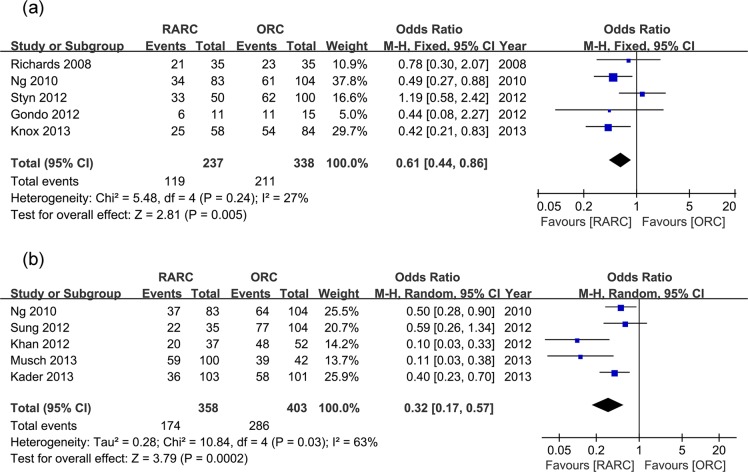
Forest plots of overall complication rates. (a) within 30 days; (b) within 90 days.

Pooled data of five studies that assessed overall perioperative complications within 30 days in 575 patients showed significantly lower complication rate in the RARC group (OR: 0.61; 95% CI, 0.44–0.86; p = 0.005) (Table 2 and Fig 2). Overall perioperative complications within 90 days were available in 761 patients from other five studies, the pooled analysis of which also showed significantly lower complication rate in the RARC group (OR: 0.32; 95% CI, 0.17–0.57; p = 0.0002) (Table 2 and Fig 2).

**Table 2:** Complication rates comparing robot-assisted radical cystectomy with open radical cystectomy.

Complication rates	No. of studies [reference]	No. of patients RARC/ORC	OR	95% CI	P value*	Study heterogeneity			
						χ^2^	df	I^2^,%	P value*
Overall within 30 d	5 [10,12,13,15,24]	237/338	0.61	0.44, 0.86	**0.005**	5.48	4	27	0.24
Grade 1 within 30 d	4 [10,12,13,24]	187/228	0.66	0.39, 1.13	0.13	2.36	3	0	0.50
Grade 2 within 30 d	4 [10,12,13,24]	187/228	0.45	0.12, 1.65	0.23	20.97	3	86	**0.0001**
Grade 3 within 30 d	4 [10,12,13,24]	187/228	0.61	0.20, 1.92	0.40	9.82	3	69	**0.02**
Grade 4 within 30 d	4 [10,12,13,24]	187/228	0.34	0.12, 0.95	**0.04**	1.10	2	0	0.58
Grade 5 within 30 d	4 [10,12,13,24]	187/228	0.46	0.12, 1.76	0.25	2.42	2	17	0.30
Overall within 90 d	5 [10,14,16,23,25]	358/403	0.32	0.17, 0.67	**0.0002**	10.84	4	63	**0.03**
Grade 1 within 90 d	3 [10,16,25]	218/308	0.81	0.12, 5.63	0.83	17.05	2	88	**0.0002**
Grade 2 within 90 d	3 [10,16,25]	218/308	0.31	0.08, 1.25	0.10	21.16	2	91	**<0.0001**
Grade 3 within 90 d	3 [10,16,25]	218/308	0.42	0.25, 0.70	**0.001**	0.31	2	0	0.86
Grade 4 within 90 d	3 [10,16,25]	218/308	0.22	0.08, 0.62	**0.004**	0.24	2	0	0.89
Grade 5 within 90 d	3 [10,16,25]	218/308	0.45	0.12, 1.66	0.23	2.99	2	33	0.22

RARC = robot-assisted radical cystectomy; ORC = open radical cystectomy; OR = odds ratio; WMD = weighted mean difference; CI = confidence interval.

* Statistically significant results are shown in bold.

**Fig 3:**
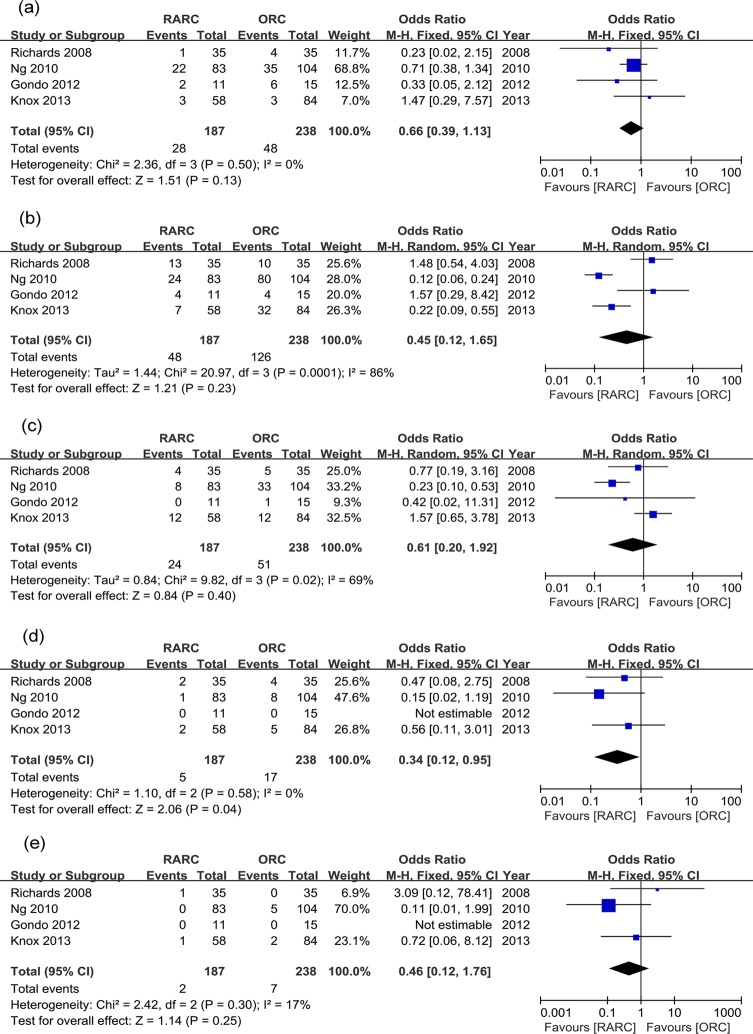
Forest plots of perioperative complication rates divided into Clavien grade 1–5 within 30 days. (a) grade 1; (b) grade 2; (c) grade 3; (d) grade 4; (5) grade 5.

**Fig 4:**
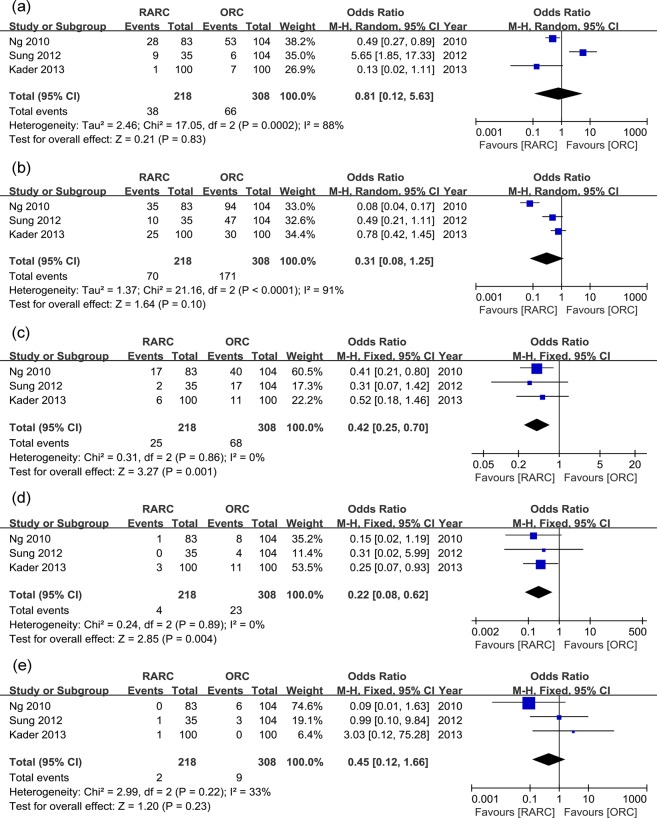
Forest plots of perioperative complication rates divided into Clavien grade 1–5 within 90 days. (a) grade 1; (b) grade 2; (c) grade 3; (d) grade 4; (5) grade 5.

Overall perioperative complications were further divided into grade 1 to 5 in studies with detailed data. Within 30 days, there was no significant difference in grade 1, 2 and 3 complication rates between two groups (p = 0.13, 0.23, and 0.40, respectively), but significantly lower grade 4 complication rate were observed in the RARC group (p = 0.04) (Table 2 and Fig 3). Within 90 days, there was no significant difference in grade 1 and 2 complication rates (p = 0.83 and 0.10, respectively), but grade 3 and 4 complication rates were significantly lower in the RARC group (p = 0.001 and 0.004, respectively) (Table 2 and Fig 4). Perioperative mortality (grade 5 complication) rates within 30 days and 90 days were similar between the two groups (p = 0.55 and 0.23, respectively) (Table 2, Fig 3 and Fig 4).

#### 2. PSM and LNY

**Fig 5:**
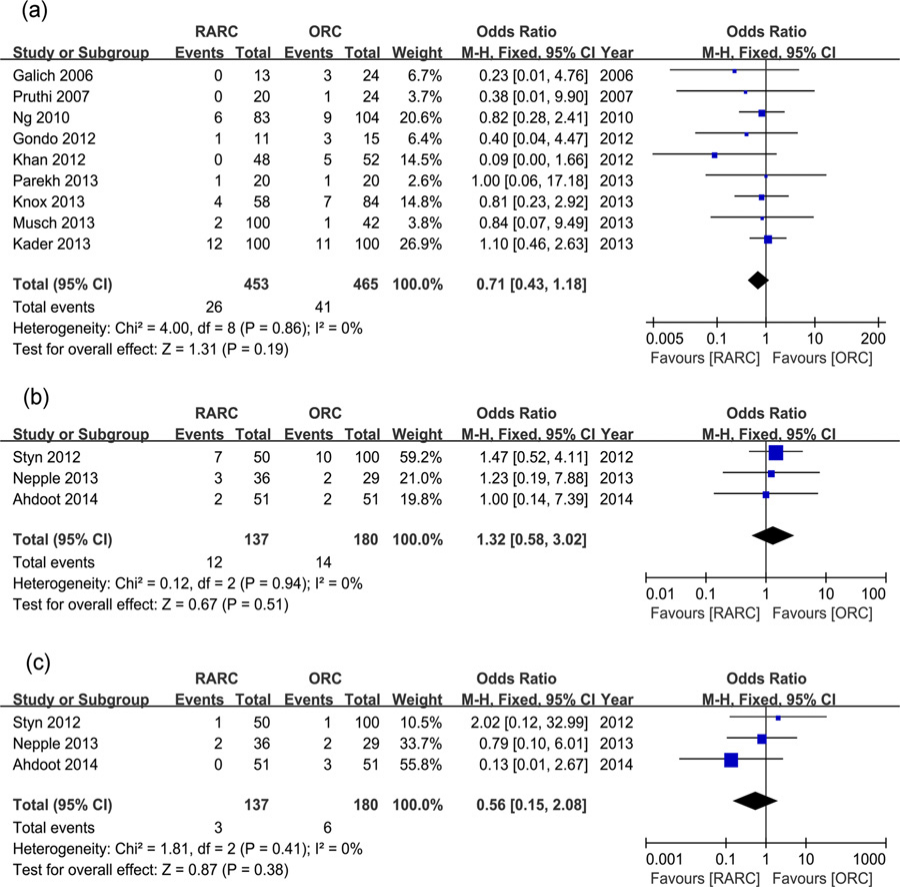
Forest plots of positive surgical margin rates.

**Fig 6:**
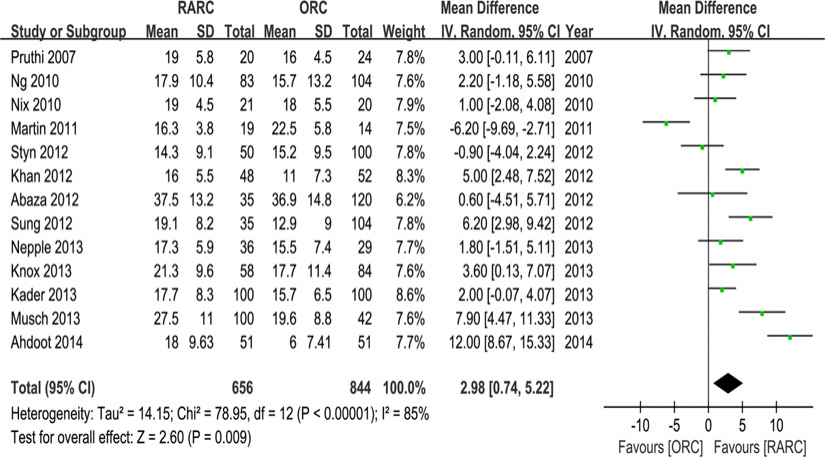
Forest plots of lymph nodes yields.

Pooled data of nine studies that assessed overall PSM rates in 918 patients showed no significant difference between RARC and ORC groups (p = 0.19) (Table 3 and Fig 5). Urethral/ureteric and soft tissue PSM rates were assessed in three studies, and there was no significant differences between the two groups (p = 0.51 and 0.38, respectively) (Table 3 and Fig 5). Pooling data of thirteen studies that counted LNY in 1500 patients showed significantly more LNY in RARC than the ORC group (WMD: 2.98; 95% CI, 0.74–5.22; p = 0.009) (Table 3 and Fig 6).

**Table 3:** Perioperative outcomes comparing robot-assisted radical cystectomy with open radical cystectomy.

Outcomes	No. of studies [references]	No. of patients RARC/ORC	OR/WMD^†^	95% CI	P value*	Study heterogeneity			
						χ^2^	df	I^2^,%	P value*
PSM rates	9 [6,7,10,13,14,22–25]	453/465	0.71	0.43, 1.18	0.19	4.00	8	0	0.86
Ureteric/urethral PSM	3 [15,17,20]	137/180	1.32	0.58, 3.02	0.51	0.12	2	0	0.94
Soft tissue PSM	3 [15,17,20]	137/180	0.56	0.15, 2.08	0.38	1.81	2	0	0.41
LNY	13 [7,10,11,14–20,23–25]	656/844	2.98^†^	0.74, 5.22	**0.009**	78.95	12	85	**<0.00001**
OT, min	9 [7,8,10,14–16,18,23,25]	474/573	73.92^†^	37.18, 110.67	**<0.0001**	91.08	8	91	**<0.00001**
EBL,10 ml	10 [7,8,10,14–16,18,23–25]	532/657	-47.39^†^	-65.13, -29.65	**<0.00001**	72.37	9	88	**<0.00001**
Perioperative transfusion	7 [8,14,15,22–25]	395/431	0.14	0.06, 0.36	**<0.0001**	30.04	6	80	**<0.0001**
Intraoperative transfusion	3 [13,15,17]	97/144	0.14	0.06, 0.34	**<0.0001**	0.37	2	0	0.83
LOS, d	10 [8,10,14–16,18,20,23–25]	563/684	-3.16^†^	-4.84, -1.48	**0.0002**	41.90	9	79	**<0.00001**

RARC = robot-assisted radical cystectomy; ORC = open radical cystectomy; OR = odds ratio; WMD = weighted mean difference; CI = confidence interval; PSM = positive surgical margin; LNY = lymph node yields; OT = operative time; EBL = estimated blood loss; LOS = length of stay.

^†^ WMD

* Statistically significant results are shown in bold

#### 3. OT, EBL, transfusion and LOS

**Fig 7:**
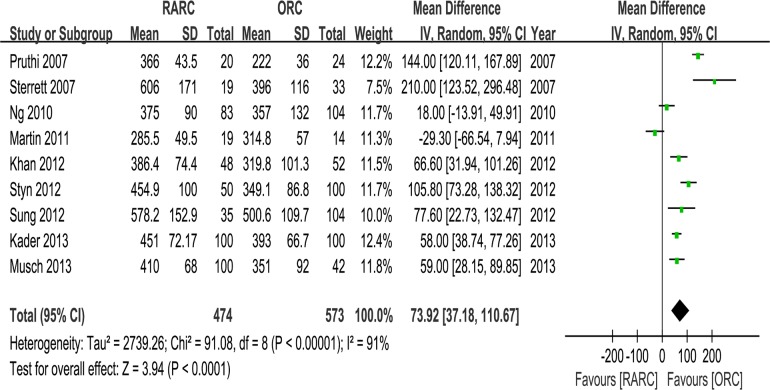
Forest plots of operative time.

**Fig 8:**
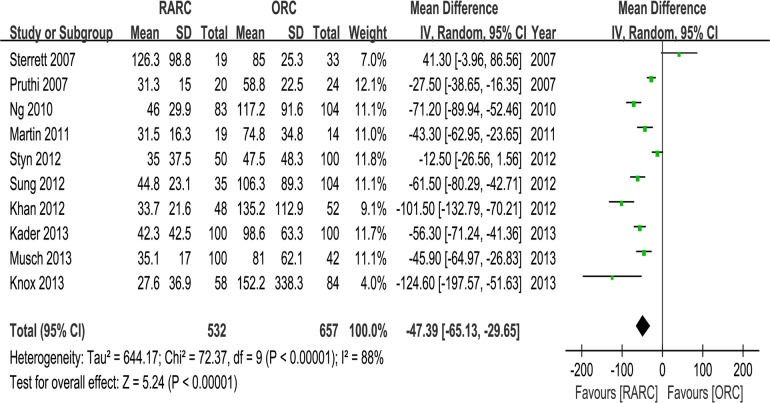
Forest plots of estimated blood loss.

**Fig 9:**
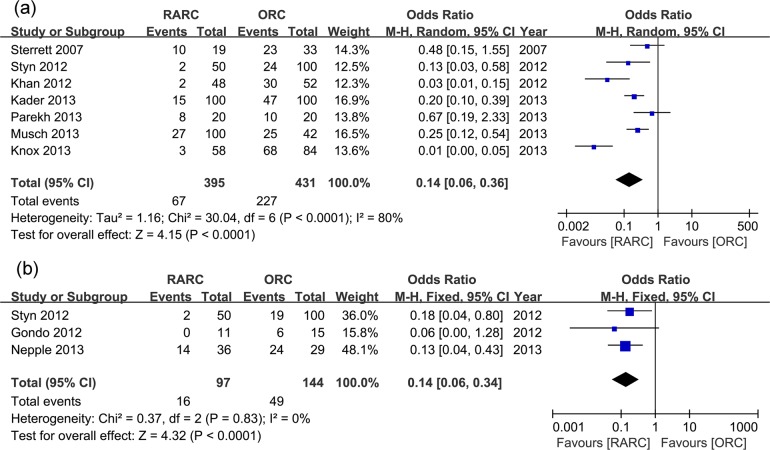
Forest plots of transfusions. (a) perioperative transfusion; (b) intraoperative transfusion.

**Fig 10:**
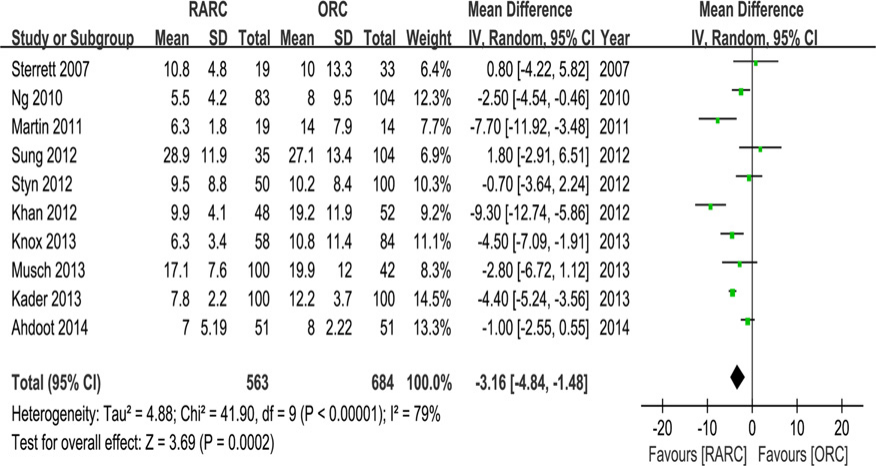
Forest plots of length of stay.

Pooled data of nine studies including 1047 patients showed significantly longer OT in the RARC than the ORC group (WMD: 73.92; 95% CI, 37.18–110.67; p < 0.0001) (Table 3 and Fig 7). Pooled data of ten studies including 1189 patients that evaluated EBL showed significantly lower blood loss in the RARC than the ORC group (WMD: -47.39; 95% CI, -65.13 –-29.65; p < 0.00001) (Table 3 and Fig 8). Pooed data of seven studies including 826 patients that evaluated perioperative transfusion rates showed significantly lower rate in the RARC than the ORC group (p < 0.0001) (Table 3 and Fig 9). Pooed data of three studies including 241 patients that evaluated intraoperative transfusion rates showed significantly lower rate in the RARC than the ORC group (p < 0.0001) (Table 3 and Fig 9). Pooled data of ten studies including 1247 patients that evaluated LOS showed significantly shorter LOS in the RARC group (WMD: -3.16; 95% CI, -4.84 –-1.48; p < 0.0001) (Table 3 and Fig 10).

#### 4. Subgroup analysis

**Fig 11:**
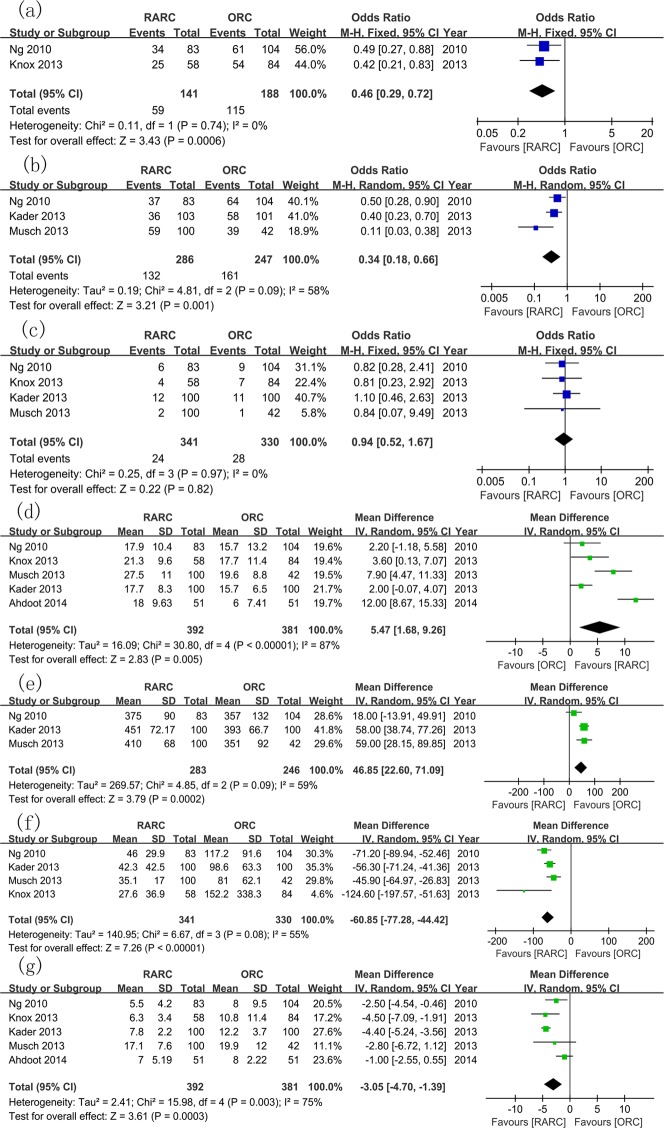
Subgroup analysis including studies with more than 50 robotic cases. (a) overall complication rates within 30 days; (b) overall complication rates within 90 days; (c) positive surgical margin rates; (d) lymph nodes yields; (e) operative time; (f) estimated blood loss; (g) length of stay.

Five studies with more than 50 RARC cases were included for subgroup analysis [10,20,23–25] (Fig 11). It continued to demonstrate lower overall complication rates within 30 days (WMD: 0.46; 95% CI, 0.29–0.72; p = 0.0006) and 90 days (WMD: 0.34; 95% CI, 0.18–0.66; p = 0.001) in RARC group. No significant difference in PSM rates between two groups was observed (WMD: 0.94; 95% CI, 0.52–1.67; p = 0.82). More LNY (WMD: 5.47; 95% CI, 1.68–9.26; p = 0.005), longer OT (WMD: 46.85; 95% CI, 22.60–71.09; p = 0.0002), less EBL (WMD: -60.85; 95% CI, -77.28 –-44.42; p < 0.00001), and shorter LOS (WMD: -3.05; 95% CI, -4.70 –-1.39; p = 0.0003) were observed in the RARC group.

#### 5. Publication bias

**Fig 12:**
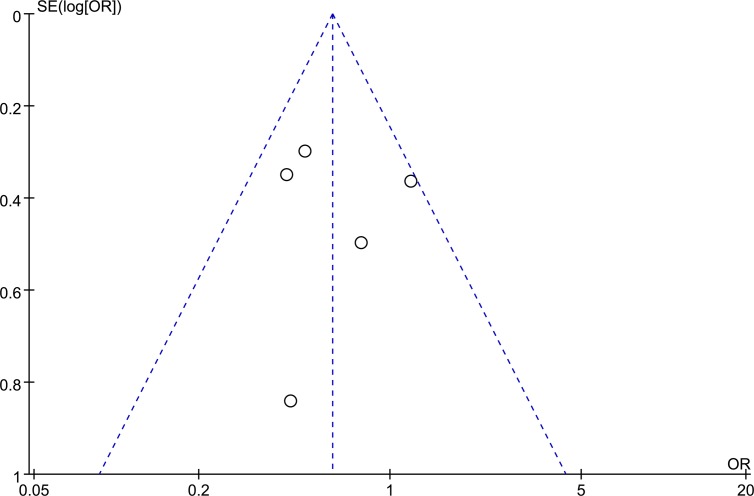
Funnel plots of the studies included in this meta-analysis reporting overall complication rates within 30 days.

Fig 12 shows funnel plots of the studies included in this meta-analysis reporting perioperative complication rates within 30 days. All studies lie inside the 95% CIs, with an even distribution around the vertical, indicating no obvious publication bias.

## Discussion

Currently, RARC are being increasingly adopted for the treatment of muscle invasive and high risk non-muscle invasive bladder cancer. Li et al. [21] conducted a systematic review and meta-analysis in 2012, with the goal of comparing RARC with ORC in terms of perioperative surgical and oncologic outcomes. However, in that study, the authors did not take fully account of the complication grades and postoperative period. Besides, some high-quality studies comparing RARC with ORC have been reported since 2012. We thus performed an updated systemic review and meta-analysis. In this review, nineteen studies, including two RCTs, ten prospective and seven retrospective studies, were included. Pooled data indicated significantly lower overall perioperative complication rates within 30 days and 90 days, more LNY, longer OT, less EBL, lower perioperative and intraoperative transfusion rates, and shorter LOS in the RARC than the ORC group.

RC is a highly challenging operation with relatively high risk of perioperative morbidity and mortality. Accordingly, perioperative complication rate is an important evaluation indicator for this procedure. As the reporting methods of complications were various and nonstandardized among the included studies, especially the postoperative period, we thus pooled data of complication rates within 30 days and 90 days postoperatively. Moreover, we also analyzed the complication rates using the Clavien grading system. Overall perioperative complication rates within 30 days and 90 days were significantly lower in the RARC group. After grading complications, grade 4 complication rate was significantly lower in the RARC group within 30 days, and grade 3–4 complication rates were significantly lower in the RARC group within 90 days. The results indicates that RARC might be safer compared to ORC, especially in the long term. Of all the included studies, Ng et al. [10] reported their complications using a more standard method with detailed data. Their results showed that RARC was an independent predictor of fewer overall and major complications within 30 days and 90 days postoperatively, and higher American Society of Anesthesiologists (ASA) score (3–4) as well as longer surgical time were independent predictors of major complications [10]. The lower complication rates in RARC group may be related to lower EBL and minimally invasive approach. Given that patients with muscle invasive bladder cancer are often elderlies with comorbidities, the lower complication rates may suggest that RARC is a better modality selection. Phillips et al. [34] concluded that RARC should be considered for patients over the age of 80 with clinical indications for RC and complication rates were acceptable even in patients with multiple comorbidities as well as those with previous abdominal surgery or pelvic radiation. Knox et al. [24] reported that RARC was superior in patients older than 70 even when compared to a younger cohort undergoing ORC. Several other studies also showed that RARC can be offered as treatment option in selected older patients [35,36].

With regard to oncologic outcomes, like most of the included studies, we used PSM rates and LNY to evaluate efficacy of RARC and ORC. PSM rate of RC are associated with progression to metastatic disease and unfavorable survival in patients with muscle invasive bladder cancer [37,38]. Our study showed that there was no difference between RARC and ORC in terms of overall unspecified PSM rates, urethral/ureteric and soft tissue PSM rates. The overall PSM rates in RARC and ORC groups were 5.7% and 8.8%, respectively, which were comparable with other RARC and ORC series [39,40]. A propensity score matched analysis showed that RARC had a lower soft tissue PSM rate compared to ORC [20]. We should cautiously draw a conclusion like RARC having equivalent PSM rate compared to ORC because PSM was highly correlated with pathological stage and selection bias did exist in some of the included studies.

Another important indicator of surgical quality of RC is LNY. Although more lymph nodes were yielded in RARC from our meta-analysis, two RCTs showed no significant difference between the two techniques. So we can only draw a conclusion of the noninferiority of robotic approach regarding lymph node dissection. Indeed, LNY in RARC is correlated with various factors, like surgeon volumes, institution volumes [41] and learning curve [42]. The data from International Robotic Cystectomy Consortium (IRCC) showed robot-assisted lymph node dissection can achieve similar LNY to those of open lymph node dissection after RC [41]. Davis et al. [43] concluded that robot assisted pelvic lymph node dissection yielded of 93% of that of open surgery by using second look open dissection method. In summary, with the three dimensional visualization and flexible instruments, RARC is expected to achieve similar oncologic outcomes to ORC after the learning curve.

The longer OT in the RARC group may due to the initial small surgeon volumes and learning curve impact. With the increasing surgical skills and better team cooperation, the OT of RARC is expected to be diminished in some degree. Our subgroup analysis indirectly suggested that OT difference between ORC and RARC was smaller for robotic cases more than 50. Several included studies reported that OT of RARC decreased when surgeon volumes increased [7,15,16]. The longer OT in RARC group can also be attributed to the extra operative steps associated with the trocar placement, docking and undocking of the robot, and conversion to extracorporeal urinary diversion [23]. Moreover, RC itself is a time-consuming operation. In the largest comparative study, the mean OT in the RARC group and ORC group were 6.25 hours and 5.95 hours, respectively [10]. Small OT gap between RARC and ORC may be not as important as other outcome differences. Besides, the lower complication rates, lower EBL as well as shorter LOS in the RARC group could easily balance the small difference with the OT.

This meta-analysis showed significant less EBL and a lower need for transfusion in the RARC group. The markedly lower blood loss in the RARC group can probably be explained by the effect of the pneumoperitoneum and more precise coagulation of bleeding vessels achieved through three dimensional visual field and flexible instruments [23,44]. Learning curve also have effects on the EBL [15,45]. Less blood loss may be a valuable finding because EBL and transfusion requirements is correlated with complications [46–49].

Learning curve, which has some influences on surgical outcomes, is an essential parameter to evaluate for an emerging technique like RARC. First of all, compared to conventional laparoscopic surgery, robotic assistance greatly reduces the learning curve for minimally invasive pelvic procedures like RC [50]. Pruthi et al. [45] described the learning curve of RARC and found initial 25 cases did have longer OT and more EBL than latter cases, but no compromises were observed with regard to complications and oncologic parameters like PSM rates and LNY. RARC can be performed safely without compromising operative, postoperative, and short-term pathologic outcomes during the learning curve for surgeons who are experienced in ORC [51]. However, for younger surgeons who have not received much ORC training, it is imperative to start with easier robotic surgeries. Guru et al. [52] highly recommended mastering robot-assisted radical prostatectomy (RARP) before attempting RARC. IRCC demonstrated an acceptable level of proficiency by the 30th case for proxy measures of RARC quality [53]. This could be a guidance for younger surgeons and their trainers.

Different financial incentives for hospital discharge exist among healthcare systems or countries [23]. The significantly shorter LOS in the RARC group is worth mentioning given that most of the included studies came from USA. Shorter LOS may due to the less invasive approach and lower complication rates. However, neither of the RCTs showed any difference despite RARC group had a trend toward fewer prolonged hospitalizations (LOS greater than 5 days) [11,22]. Whether the lower complication rates and shorter LOS in the RARC group can lead to a potential benefit in quality of life (QOL) is another considerable question. One of the included RCTs reported their QOL outcomes in a different paper, in which QOL was assessed by Functional Assessment of Cancer Therapy–Vanderbilt Cystectomy Index (FACT–VCI) questionnaire preoperatively and then at 3, 6, 9 and 12 months postoperatively [22,54]. The results showed a slightly higher physical well-being score in the RARC group at 6 months, but no difference was observed in other domains [54]. Guru at al [55] reported that patients who had RARC required less opiates postoperatively compared to those who had ORC, although both groups achieved similar pain control. However, another study showed better sexual functions in the ORC group over time [56]. Given that there is still lack of standardization of QOL measures and much of the studies were retrospective or had small sample size, larger RCTs with standard outcomes are needed to characterize QOL difference between RARC and ORC.

Cost analysis between two groups, although was not one of our outcomes, deserves to be discussed because health care cost control is one of the most essential topics in modern era [57]. Smith et al. [58] concluded that RARC was associated with a higher financial cost (+$1,640) than ORC in the perioperative setting. However, they excluded the analysis of hospital medication cost, as well indirect cost of complications. Another study performed a cost-analysis between RARC and ORC using a model, which included both direct and indirect cost of 30-day complications and hospital medications [18]. Interestingly, actual total patient costs revealed a 38% cost advantage favoring RARC due to increased hospitalization costs for ORC and higher complication rates [18]. The cost benefit of RARC was also observed in the largest cohort study [10,59]. Various factors, such as hospital volume, LOS, OT, morbidity, and complexity of urinary diversion, can influence the cost. All we can expect is large RCTs between the two surgical approaches and hope they can effectively assess whether a cost benefit does exist.

Although RARC has its advantages, ORC is still an important and indispensable procedure for urologist to learn. Even in USA, which is equipped with the largest number of robotic surgical system in the world, RARC is only a small portion of total RC cases. Data from the Nationwide Inpatient Sample in 2009 to 2011 showed that RARC accounted for about 12.6% of all RC cases [60]. ORC will still be the gold standard for muscle invasive bladder cancer in the short and medium term. Like all other surgeries, ORC training can help younger surgeons to be familiar with anatomic structure directly. Besides, surgical skills and experience from open cases are essential because all surgeons have to be prepared for intraoperative conversion during any minimally invasive procedures.

There are certain limitations to be considered in the present review and meta-analysis. The main limitation is that most of the included studies were observational studies except two RCTs, both of which used inappropriate randomization method and had limited number of patients (40 and 41, respectively) [11,22]. Overall, studies included in the analysis had small patient cohorts. Ten out of nineteen studies had less than 100 patients. Secondly, some of the included studies probably had the risk of selection bias which might limit the power of this meta-analysis. However, our study has relatively large sample size and standard data extraction and analysis. Our conclusions thus could provide guidance for both urologists and healthcare policy makers.

## Conclusions

Nineteen studies assessing RARC versus ORC were included for this meta-analysis. The results indicated that RARC may be associated with lower overall perioperative complication rates within 30 days and 90 days, lower grade 3 perioperative complication rate within 30 days, lower grade 3 and 4 perioperative complication rates within 90 days, more LNY, longer OT, less EBL, lower need for transfusion and shorter LOS. Conclusively, RARC appears to be a safer, less invasive procedure with same efficacy when compared with ORC. In spite of our rigorous methodological review, limitations of the included studies imposed restrictions on us to draw definite conclusions. Large cohort studies and well-designed RCTs with longer follow-up are needed to confirm and update the findings of this analysis.

## Supporting Information

S1 PRISMA Checklist PRISMA Checklist. (DOC)
